# Dispersion-tunable low-loss implanted spin-wave waveguides for large magnonic networks

**DOI:** 10.1038/s41563-025-02282-y

**Published:** 2025-07-09

**Authors:** Jannis Bensmann, Robert Schmidt, Kirill O. Nikolaev, Dimitri Raskhodchikov, Shraddha Choudhary, Richa Bhardwaj, Shabnam Taheriniya, Akhil Varri, Sven Niehues, Ahmad El Kadri, Johannes Kern, Wolfram H. P. Pernice, Sergej O. Demokritov, Vladislav E. Demidov, Steffen Michaelis de Vasconcellos, Rudolf Bratschitsch

**Affiliations:** 1 Institute of Physics and Center for Nanotechnology (CeNTech), University of Münster, Münster, Germany; 2 Institute of Applied Physics, University of Münster, Münster, Germany; 3 Center for Soft Nanoscience, University of Münster, Münster, Germany; 4 Kirchhoff-Institute for Physics, Heidelberg University, Heidelberg, Germany

**Keywords:** Magnetic properties and materials, Spintronics, Electrical and electronic engineering, Electronic devices, Polarization microscopy

## Abstract

Magnonic networks based on magnetic insulators are poised to revolutionize information processing due to their energy efficiency. However, current experimental realizations of spin-wave waveguides, which constitute the building blocks of such a network, suffer from limited spin-wave propagation lengths and inefficient dispersion tuning capabilities. Here we realize low-loss spin-wave waveguides in yttrium iron garnet thin films using silicon ion implantation, which creates an amorphous waveguide cladding. We measure spin-wave decay lengths exceeding 100 µm in submicrometre waveguides. The dispersion of the waveguides can be continuously tuned due to the precise and localized ion implantation, which sets them apart from commonly etched waveguides. Using our maskless waveguide definition, we demonstrate a large-scale magnonic network consisting of 198 crossings, paving the way for wafer-scale magnonic integrated circuits.

## Abstract (web summary)

Silicon-ion-implanted yttrium iron garnet technology enables low-loss and dispersion-tunable magnonic waveguides with spin-wave decay lengths of >100 µm, which pave the way for large-scale, energy-efficient magnonic integrated circuits.

## Main

Spin waves are collective excitations of magnetization in magnetic materials, which present enticing prospects for next-generation information processing^1–3^. Their distinctive attributes, such as high-speed operation within the gigahertz to terahertz frequency band^4,5^ and a natural strong nonlinearity, make them particularly appealing. Recently, a notable surge has occurred in recognizing the potential of using spin waves in nanoscale magnetic structures and networks for signal processing and computing applications^6,7^. This emerging technology holds promise to overcome limitations inherent in traditional semiconductor microelectronics in terms of computational density and high-dimensional processing capacity. In particular, the low-energy footprint of spin-wave technology is highly appealing^2^.

The versatility of spin-wave technology lies in its capability to encode information within the phase, amplitude and frequency of spin waves. Similar to electromagnetic waves, this strategy enables a flexible range of data-processing operations, exploiting the dependence of propagation characteristics on these parameters. Successful demonstrations cover a diverse range of spin-wave devices, including crossings^8,9^, couplers^10^, splitters^11^, majority gates^12^, (de-)multiplexers^13^, logic gates^8,14^, interferometers^15^, spectrum analysers^16^ and memories^17^. These devices can act independently as information processing units or seamlessly integrate into complex networks with advanced functionalities^7,8,11,14–16,18–23^.

The pivotal and fundamental components linking these functional elements within a complex network are tailored waveguides for spin waves. These waveguides play a crucial role in confining and guiding spin waves from one element to another and thus require minimal propagation losses. Moreover, such waveguides and their combinations also serve themselves as functional spin-wave devices^3^.

Among the known magnetic materials, yttrium iron garnet (YIG) features the lowest damping and highest propagation length of spin waves, reaching millimetres^24,25^. To date, predominantly lithographic approaches have been employed to realize waveguides for spin waves. The state-of-the-art fabrication approach for creating nanoscale waveguides in YIG is based on reactive ion etching of thin YIG films^26,27^. However, even with state-of-the-art etching processes and high-quality YIG films, the reported maximum propagation length is 54 µm (ref. ^28^). Another emerging approach involves the fabrication of hybrid structures, wherein YIG films are combined with ferromagnetic metal nanostripes to define nanoscopic spin-wave transporting channels within YIG by dipolar coupling^29^. This technology requires additional lithography processing steps including masking, metal deposition and lift-off. Notably, spin-wave propagation lengths of ~20 µm in submicrometre waveguides have been demonstrated using this method^29^.

Recently, the manipulation of spin waves in YIG was demonstrated by ion implantation^30–32^. Focused ion beam writing with Ga and He ions has enabled the precise modification of YIG films on a submicrometre scale. By changing the effective magnetization, the magnonic index of refraction can be engineered^31,33^, allowing one to guide, focus and diffract magnons in the implanted regions^31,34^, with the drawback of increased spin-wave damping.

The lack of low-loss waveguides has prevented the fabrication of large-scale spin-wave networks, despite the demonstration of many individual elements, as mentioned above. Furthermore, although numerous theoretical proposals for spin-wave computing circuits have been put forth^3,35^, the most complex spin-wave network in YIG consists of only four crossings and eight linear waveguide ports, thus far^36^.

Here we report the maskless fabrication and characterization of spin-wave waveguides realized by precision implantation of silicon ions in a thin YIG film. By implanting outside of the area where the spin wave propagates (analogous to the cladding in optical fibres), losses remain low and the spin-wave dispersion can be tailored. We achieve a spin-wave propagation length of >100 µm. Using our etchless approach, we demonstrate an integrated spin-wave network consisting of 34 parallel input ports, 198 crossings and 34 outputs. Dispersion control is demonstrated by a spin-wave mode expander, which is realized by tailoring the implantation dose for two connected waveguides of different widths. These results pave the way for realizing advanced magnonic networks with unparalleled control and exciting avenues for realizing low-loss large-scale spin-wave computing systems.

**Fig. 1:**
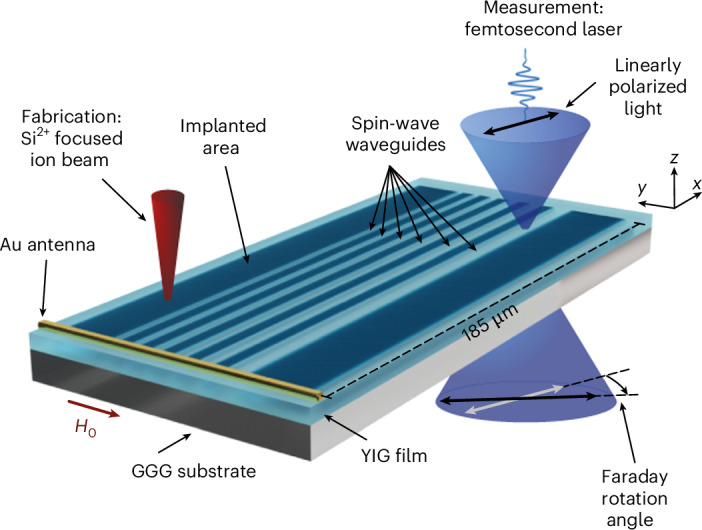
Maskless fabrication of spin-wave waveguides and optical measurement. The sample consists of a thin YIG film grown on a GGG substrate. Parallel rectangular areas (dark blue) are implanted using a Si^2+^ focused ion beam (red cone). This way, spin-wave waveguides (light blue) are formed between the implanted areas with different nominal widths ranging from 500 nm to 3 µm. To image spin-wave propagation within the waveguides, we measure the Faraday rotation of a linearly polarized femtosecond laser beam (blue) in a sample scanning microscope.

We use a commercially available 110-nm-thick YIG film grown via liquid-phase epitaxy on a 3-inch gadolinium gallium garnet (GGG) wafer of 500 µm thickness (Matesy). A gold microstrip antenna fabricated with electron-beam lithography on the YIG film (Methods) serves to excite spin waves with a continuous-wave microwave signal (Fig. 1). We apply an external static in-plane magnetic field *H*_0_ of *μ*_0_*H*_0_ = 50 mT, where *µ*_0_ is the vacuum permeability, aligned parallel to the Au antenna, to launch surface-mode spin waves^37^. For spin-wave detection, various methods exist, such as Brillouin light scattering^38^, Kerr imaging or detection via colour centres in diamond^39^ or hexagonal boron nitride^40^. Here we employ a custom-built Faraday-imaging sample-scanning microscopy set-up (Methods and Supplementary Information) based on a low-noise high-stability femtosecond fibre laser system^41^. Using a phase-locked loop, we generate the microwave frequency for spin-wave excitation synchronized with the laser repetition frequency (*f*_rep_) of 40.12 MHz. This way, we perform sweeps of the excitation frequencies in steps of integer multiples of *f*_rep_.

To create the spin-wave waveguides, we implant doubly positively charged silicon ions (Si^2+^) with a kinetic energy of 70 keV into the YIG film using a Raith Velion focused ion beam machine (Fig. 1). Details of the fabrication process are provided in Methods. Simulations with the SRIM (Stopping Range of Ions in Matter) software^42^ demonstrate that the silicon ions penetrate the entire YIG layer (Supplementary Fig. 1) and indicate a lateral spread on the same order of magnitude. Notably, their penetration depth is much larger than that of previously used Ga ions with a kinetic energy of 50 keV, which could modify only the top 25 nm of the layer^31,32^. We realize linear waveguides with nominal widths from 3 µm down to 500 nm by implanting the area outside the waveguide with three different ion doses of 2.5 pC µm^−2^, 5 pC µm^−2^ and 10 pC µm^−2^.

**Fig. 2:**
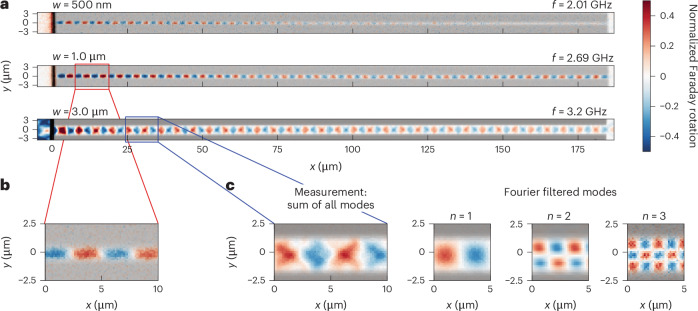
Spin-wave propagation in linear waveguides of different widths. **a**, The nominal waveguide widths (*w*) range from 500 nm (top of **a**) to 1.0 µm (middle) to 3.0 µm (bottom). The Faraday rotation images reveal spin waves (coloured), propagating for more than 100 µm. The superimposed optical transmission image visualizes the areas implanted with a dose of 10 pC µm^−2^ (dark grey) and the gold antenna (black) on the left (at *x* = 0). The spin wave is excited at the frequency *f* indicated on the right. **b**, Zoomed-in view of the measured mode profile in the 1-µm-wide waveguide, indicating single-mode spin-wave propagation (*n* = 1). **c**, Zoomed-in view of the 3-µm-wide waveguide (left) and mode decomposition (right), revealing higher-order modes (*n* = 2 and 3). Source data

Figure 2 shows the spin-wave propagation in three example waveguides of 500 nm, 1.0 µm and 3.0 µm width. The Faraday rotation signal (red-to-blue colourmap) is a measure of the dynamic out-of-plane component of the magnetization in the YIG film. The dimensions and position of the implanted regions can be readily identified in optical transmission, overlaid as a greyscale image in Fig. 2. The ion-implanted regions exhibit a lower optical transmission of the probing laser than the pristine YIG film and hence appear darker. The black vertical line at *x* = 0 μm originates from the gold antenna used for magnon excitation. Most importantly, we observe magnons propagating along the entire structure distance of 185 µm in all waveguides. At the exit of the waveguide and depending on the frequency, the magnons either continue propagating in the plain YIG film or are back reflected, leading to the formation of standing waves in the waveguide. The propagation in the narrowest waveguides is single mode (*n* = 1; Fig. 2b). By contrast, the measured spin-wave mode profiles for the wider waveguides are more complex (Fig. 2c). Decomposing the modes using spatial Fourier filtering^43^ reveals the underlying higher-order modes (*n* > 1). The appearance of the even spin-wave modes (for example, *n* = 2) is likely due to a slight tilt of the external magnetic field of a few degrees with respect to the gold antenna and waveguide structures^43^.

To study the spin-wave propagation in more detail, we record multiple Faraday rotation images for excitation frequencies between 1.9 GHz and 4 GHz and extract the dispersion relation, the decay length and the mode profile of the confined spin waves in the waveguides.

From the measured decay of the Faraday rotation amplitude, we extract the frequency-dependent decay length of the spin-wave amplitude. For an accurate determination, we take the appearance of standing waves due to the reflection at the end of the waveguide in our fitting model into account. The largest measured decay lengths of the spin-wave amplitude are observed for waveguides fabricated with the lowest implantation dose of 2.5 pC µm^−2^. The decay length exceeds 100 µm for all waveguide widths, demonstrating the low-loss performance of the concept. A detailed analysis of the frequency-dependent decay lengths for all waveguide widths is presented in Supplementary Fig. 6.

**Fig. 3:**
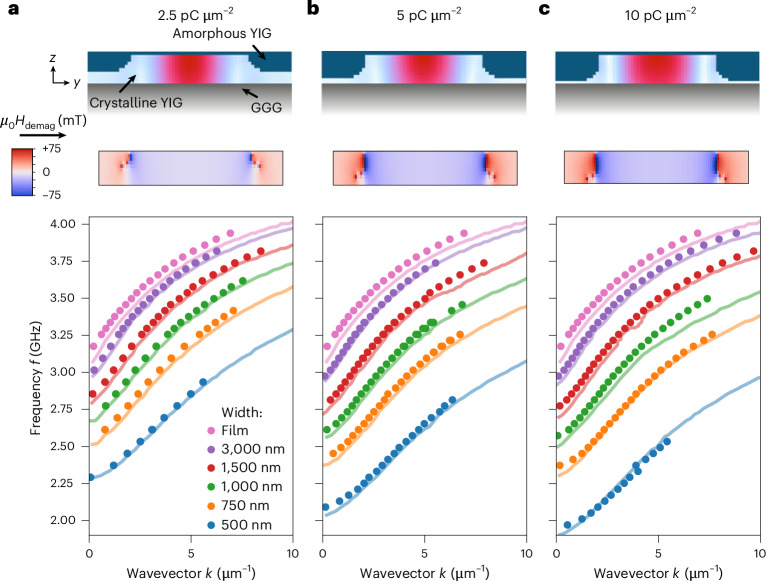
Spin-wave dispersion relation and confinement. **a**–**c**, Dispersion relations for spin-wave waveguides of different widths, created with three different implantation doses (2.5 (**a**), 5 (**b**) and 10 pC µm^−2^ (**c**)) and measured at an external magnetic field of 50 mT. The cross-sections of the waveguides on top depict the distribution of amorphous and crystalline YIG for the different implantation doses, respectively. Dark blue areas denote amorphous YIG, while the rest is crystalline YIG. The superimposed heatmap visualizes the spatial distribution of the first waveguide mode (*n* = 1). With increasing dose, a larger part of the YIG gets amorphized, causing a downshift in frequency of the spin-wave dispersion relations. The negligible saturation magnetization of the amorphous regions gives rise to a demagnetizing field (*H*_demag_) in the waveguide (middle images), whose magnitude depends on the depth of the amorphized YIG. Coloured solid lines in the bottom images denote mumax^3^ simulations of the waveguide structures. The width of the simulated waveguides is set 100 nm smaller than the nominal waveguide width to account for the effective narrowing of the waveguide due to the implantation profile of the ions. Source data

The dispersion relations of the fundamental (*n* = 1) waveguide modes (Fig. 3) clearly differ from the dispersion of the pristine YIG film and are shifted to lower frequencies. The shift becomes stronger the narrower the waveguides are. Scanning transmission electron microscopy (STEM) measurements (Supplementary Fig. 3) visualize the cross-section of the waveguides (Fig. 3, top), showing that the ion implantation leads to YIG amorphization. The amorphous regions extend from the sample surface towards the GGG substrate below and form a curved profile with a thin remaining crystalline YIG pedestal at the edges of the waveguide (Fig. 3, top). The thickness of the amorphous YIG region increases with increasing implantation dose^44^, reaching a fraction of 90% of the total YIG thickness at an implantation dose of 10 pC µm^−2^.

To understand the spin-wave confinement, we perform three-dimensional numerical micromagnetic simulations with the software mumax^3^ for different waveguide widths and varying thicknesses of the amorphized layer^45^. We model the cross-sectional shape of the amorphized regions according to the STEM images. The saturation magnetization *M*_sat_ of amorphous YIG is only 1% of that of crystalline YIG^46^. This reduced magnetization in the implanted areas on both sides of the crystalline YIG waveguide causes demagnetization effects, reducing the effective magnetic field inside the waveguide^47^. Notably, for amorphization depths larger than 20–30%, the demagnetizing field is nearly uniform along the out-of-plane direction *z* in the centre of the waveguide (Fig. 3 and Supplementary Section 5). As a result of the decreased effective magnetic field in the waveguide, the dispersion relation of spin waves in the waveguide is shifted substantially towards lower frequencies. This shift causes a frequency and momentum mismatch between spin waves inside and outside of the waveguide region and creates the confinement.

Using amorphous layer thicknesses of 60%, 80% and 90% of the total waveguide thickness, we can numerically reproduce the measured dispersion curves for the different waveguide widths and implantation doses (Fig. 3a–c). The width of the waveguides in the mumax^3^ simulation is diminished by 100 nm compared with the design of the implantation pattern due to the lateral implantation profile of the ions (Supplementary Fig. 1), which effectively narrows the width of the waveguides. SRIM simulations clearly show a lateral spread of about 50 nm from the centre of the ion beam (Supplementary Fig. 1), which is also verified by the STEM measurements of the cross-section of the waveguide (Supplementary Fig. 3a–c). Thus, the total width of the waveguide is reduced by 100 nm, because the narrowing is present on both edges of the waveguide.

**Fig. 4:**
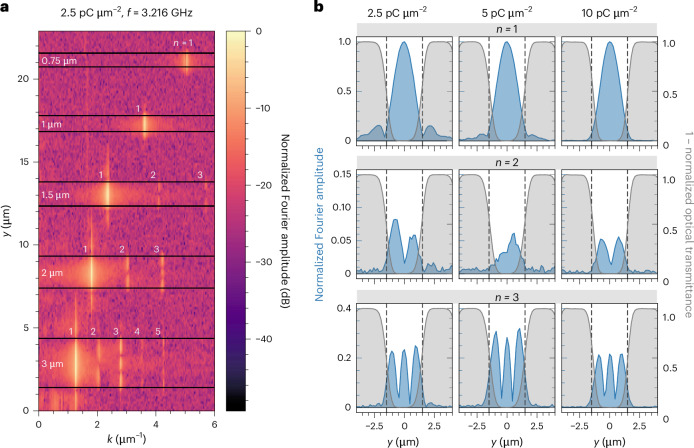
Transverse spin-wave modes in the waveguides. **a**, Line-by-line spatial Fourier transform of the normalized Faraday rotation image using a logarithmic colour coding. Waveguides are marked by black lines and highlighted with their nominal widths (750 nm to 3 µm). Higher-order transverse spin-wave modes are visible in the wider waveguides (2.5 pC µm^−2^ dose) and indicated with numbers *n*. The modes extend into the adjacent implanted areas. **b**, Transverse profile of the Fourier transform of the 3-µm-wide waveguide for individual modes (*n* = 1, 2, 3) and three implantation doses (2.5 pC µm^−2^, 5 pC µm^−2^, 10 pC µm^−2^), normalized to the *n* = 1 mode for each dose (blue). The superimposed profiles (1 – normalized optical transmittance, shown in grey) visualize the implanted areas outside the waveguide. The nominal waveguide width is indicated by dashed vertical lines. At lower implantation doses, the modes extend more into the adjacent implanted areas. Source data

The Faraday rotation images also reveal the lateral confinement and the transverse mode profile of the spin waves in the fabricated waveguides. Figure 4a shows a line-by-line spatial Fourier transform of the Faraday rotation image of five waveguides of nominal widths of 750 nm (top) to 3 µm, recorded at a frequency of 3.216 GHz. Narrower waveguides below 1 µm support a single mode with *n* = 1, while wider ones also host higher-order modes up to *n* = 5. Furthermore, the Fourier images (Fig. 4a and Supplementary Fig. 7) reveal that spin waves extend evanescently into the remaining crystalline film pedestal below the amorphous YIG adjacent to the waveguide. In Fourier space, we can now filter each individual mode and extract the corresponding transverse intensity profile. In Fig. 4b, the intensity profiles for the modes *n* = 1–3 in the 3-µm-wide waveguide are shown for the three different implantation doses. As expected, the intensity of the higher-order modes is notably lower than that of the fundamental *n* = 1 mode. The width of the waveguide can be identified from the overlaid optical transmission profile (shaded area). For low implantation doses of 2.5 pC µm^−2^ and 5 pC µm^−2^, we observe a considerable spin-wave amplitude in the implanted barriers (particularly for the *n* = 1 mode). For the highest implantation dose of 10 pC µm^−2^, the spin-wave amplitude is almost absent in the implanted areas. This effect is due to a stronger confinement of the spin wave resulting from the reduced thickness of the remaining crystalline film pedestal at higher implantation doses.

**Fig. 5:**
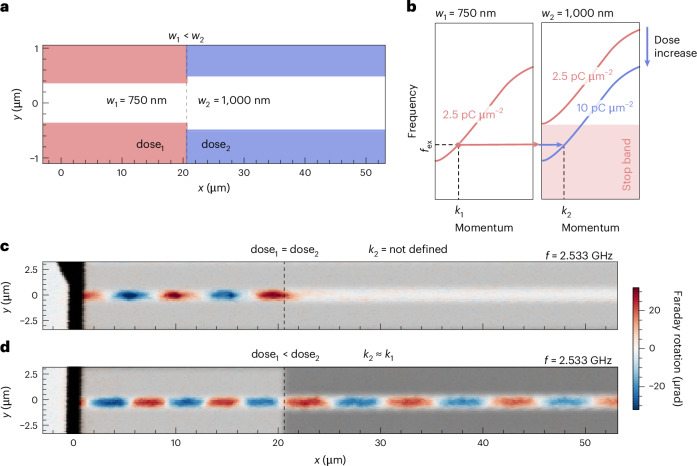
Dispersion engineering by ion implantation with different doses. **a**, Schematic illustration of the investigated structure, consisting of two connected spin-wave waveguides of different widths (*w*_1_ and *w*_2_). A 750-nm-wide waveguide is directly connected to a 1,000-nm-wide waveguide, formed by ion-implanted areas with dose_1_ and dose_2_, respectively. **b**, Schematic dispersion relations of spin waves propagating in 750 nm (left) and 1,000 nm (right) waveguides with implantation doses of 2.5 pC µm^−2^ and 10 pC µm^−2^. At frequency *f*_ex_ a spin wave can be excited in the narrow 750 nm waveguide formed with implantation doses of 2.5 pC µm^−2^ (left). However, for the wider 1,000 nm waveguide, no mode exists at this frequency (stop band). By changing the implantation to a higher dose, the stop band can be tuned to compensate for dimensional changes and match the wavevectors *k*_1_ and *k*_2_. **c**,**d**, Faraday rotation images visualizing the spin-wave propagation. The superimposed optical transmission images indicate the implantation dose (greyscale). In **c**, dose_1_ and dose_2_ are equal (2.5 pC µm^−2^), resulting in strongly suppressed spin-wave propagation in the wider (1,000 nm) waveguide and strong back reflections. In **d**, dose_2_ (10 pC µm^−2^) is chosen to allow spin-wave propagation, resulting in a similar wavelength in both parts and spin-wave propagation across the junction. Source data

A distinctive advantage of our implantation method lies in its capability to engineer the dispersion characteristics with two degrees of freedom: waveguide width and implantation dose. To demonstrate this unique feature, we fabricated a spin-wave beam expander structure that consisted of two connected linear spin-wave waveguides of different widths (750 nm and 1 µm), defined by ion implantation using two distinct doses (referred as dose_1_ and dose_2_). The design is illustrated schematically in Fig. 5a. Notably, when applying equal implantation doses of 2.5 pC cm^−2^, pronounced back reflections occur, and the spin-wave propagation is completely suppressed in the wider 1 µm waveguide. However, by tuning the relative dosage such that dose_1_ < dose_2_, spin-wave transmission across the junction can be restored (Fig. 5d). This effect can be explained by analysing the respective dispersion relations depicted in Fig. 5b. For the 750 nm waveguide, the dispersion relation of the waveguide with an implantation dose of 2.5 pC µm^−2^ (red line) crosses the excitation frequency of *f*_ex_ indicated by the dashed line, which means that a confined spin-wave mode for this particular frequency exists. In the 1-µm-wide waveguide, by contrast, the spin-wave gap becomes larger (that is, the dispersion relation shifts towards higher frequencies) and thus prevents the spin-wave propagation for the same lower implantation dose. As a result, the spin wave is reflected at the junction, as seen in Fig. 5b. By increasing the implantation dose (dose_2_), we can shift the dispersion relation towards lower frequencies, allowing for spin-wave modes in the 1-µm-wide waveguide at the frequency *f*_ex_. As a consequence, the spin-wave transverses the junction. Thus, it is possible to tune the dispersion in linear spin-wave waveguides to compensate for geometric changes. This could be of particular interest for the optimization of waveguide intersections. Another further example tailoring spin-wave wavelengths via implantation of the waveguide cladding is described in Supplementary Information (Supplementary Fig. 8).

**Fig. 6:**
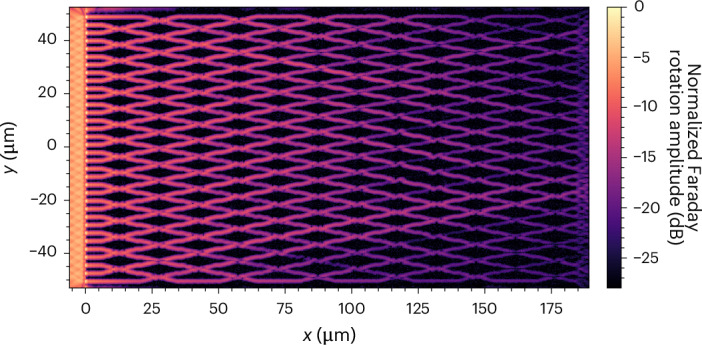
Spin-wave network. The network consists of 34 parallel input ports, 198 waveguide crossings and 34 output ports. The Faraday rotation image reveals spin-wave propagation across the network for 185 µm. Source data

To demonstrate the potential of the new spin-wave waveguide technology for integrated circuits, we fabricate a spin-wave network with 34 parallel input ports, 198 waveguide crossings and 34 output ports (Supplementary Fig. 9 for optical transmission image). In Fig. 6 we present the spin-wave amplitude determined from a Faraday rotation image of the large-scale network, which shows propagation across the entire network with a total length of 185 µm.

In conclusion, we have demonstrated low-loss spin-wave waveguides realized by the implantation of silicon ions in a thin YIG film. We verified spin-wave decay lengths exceeding 100 µm and tailored dispersion tuning. Using the new spin-wave waveguide technology, we demonstrated a spin-wave network with 198 crossings. The maskless implantation process allows for the fabrication of multiple tailored spin-wave structures on a single substrate and can be scaled up to create wafer-size magnonic integrated circuits. Hence, the presented approach paves the way for realizing low-loss large-scale spin-wave computing systems.

## Methods

### Electron-beam lithography of antennas

We use a commercially available 110-nm-thick YIG film on a 3-inch GGG wafer, obtained from Matesy. Initially, we dice the wafer into smaller 10 × 10 mm pieces using a Disco DAD3221 dicing saw. To reduce dust contamination during the dicing process, the wafer is coated with a thick layer of photoresist. The cut pieces are cleaned in acetone and isopropanol in an ultrasonic bath and then put on a hotplate at 150 °C for 4 min. Subsequently, an adhesion promoter (AR 300-80 new, Allresist) is spin-coated at 4,000 rpm for 60 s and baked for 5 min at 150 °C, followed by poly(methyl methacrylate) (PMMA) resist for electron-beam lithography (AR-P 679.04, Allresist), which is spin-coated at 4,000 rpm for 60 s and baked for 2 min at 180 °C. Since YIG and GGG are insulating materials, a conductive polymer (AR-PC 5090, Allresist) is finally spin-coated (4,000 rpm for 60 s and baked for 2 min at 90 °C) to remove excess charges during electron-beam exposure. The resist is structured using a Raith EBPG5150 machine with a voltage of 100 kV. A beam current of 5 nA is used for the microstrip Au antennas and 100 nA for larger structures, with a dose of 600 µC cm^−2^. Additionally, marker structures are written during this step, which are later used during focused ion beam implantation. After electron-beam exposure, the sample is rinsed with deionized water to remove the conductive polymer AR-PC 5090 and then developed using a 1:3 mixture of methyl isobutyl ketone and isopropanol for 120 s. In the next step, a seed layer of 5 nm Cr followed by a Au layer of 125 nm are deposited on the sample in a homebuilt electron-beam physical vapour deposition machine. Finally, the lift-off is done using acetone. The resulting Au microstrip antennas have a width of 1.2 µm and a height of 125 nm, as measured by an atomic force microscope, with lengths ranging from 40 µm to 100 µm. They are connected with a 20-µm-wide wire to larger contact pads used for wire bonding and electrical excitation.

### Focused ion beam implantation

To alter the magnetic properties of the thin YIG film, silicon ions are implanted into the sample. The implantation process is carried out using a Raith Velion machine. Similar to electron-beam lithography, a conductive layer (AR-PC 5092, Allresist) is spin-coated (4,000 rpm for 60 s and 2 min at 90 °C) on top of the insulating YIG to remove excess charges. After the ion beam implantation, the sample is cleaned with deionized water to remove the polymer. For the implantation, doubly charged silicon ions (Si^2+^) are accelerated with a voltage of 35 kV, resulting in a kinetic energy of the ions of 70 keV. We use a 20 µm aperture and a beam current of 12 pA during the exposure. To create different implantation doses, the pixel dwell time is varied. Using four Au micrometre-sized markers fabricated in the previous electron-beam lithography step, it is possible to precisely align the Si^2+^ ion beam in a write field of 200 µm × 200 µm. In that way, we are able to implant next to the Au antenna at a distance of only 250 nm without any visible damage to the antenna structure. The linear spin-wave waveguides in the YIG film are defined by two rectangular areas with a length of 185 µm and a width of 3 µm, which are implanted with a dose of 2.5 pC µm^−2^, 5 pC µm^−2^ or 10 pC µm^−2^ (Fig. 2). Waveguides of different width are fabricated by varying the distance between these two implanted rectangular structures. We create spin-wave waveguides of 185 µm length and nominal design widths of 3 µm, 2 µm, 1.5 µm, 1 µm, 750 nm and 500 nm. The ions are implanted in the vertical direction of the YIG film resulting in a cone shaped implantation profile (Supplementary Fig. 1 for a simulation). This effect becomes visible for the waveguides of smallest widths; that is, the real widths are smaller than the nominal widths. The ion implantation leads to a small swelling of the implanted areas on the order of nanometres to a few tens of nanometres depending on the ion dose^48^. An atomic force microscope image of the implanted areas of the sample, recorded after all other measurements had been performed, yielded a value of 2 nm.

### Faraday rotation imaging

To visualize spin waves, we employ a custom-built Faraday-rotation sample-scanning microscope, detailed in Supplementary Fig. 2. Spin waves are excited using Au antennas directly integrated onto the YIG surface, powered by continuous-wave microwave signals. The microwaves are generated by upscaling the laser repetition frequency via a phase-locked-loop synthesizer, ensuring synchronization with the laser pulses for the stroboscopic measurement of the spin waves. Through precise adjustment of the phase relationship between laser pulses and microwaves, the spin-wave dynamics can be resolved. In the surface mode (Damon–Eshbach) geometry used here, spin waves induce an out-of-plane magnetization component, affecting the polarization state of transmitted laser light via the magneto-optic Faraday effect. Our detection set-up features a femtosecond fibre laser system operating at a wavelength of 480 nm, with a pulse width of approximately 200 fs and a repetition rate of 40.12 MHz (ref. ^41^). Initially, a polarizer determines the linear polarization of the laser beam. The beam is then focused onto the sample, mounted on a piezo scanning stage, via a ×100 Nikon TU Plan objective lens with a numerical aperture of NA = 0.9. The transmitted beam is collected by a ×50 objective lens (Nikon TU Plan ELWD, NA = 0.6) and passes through a half-wave plate to rotate the polarization to 45° relative to the subsequent Wollaston prism. To analyse the polarization state of the transmitted light, a photodiode with two channels (A and B) is employed, measuring A – B and A + B. To isolate the spin-wave-induced polarization change, the driving microwave signal is modulated at a frequency of 80 kHz, and the optical signal is detected at the same frequency using a lock-in amplifier (Stanford Research Systems SR830). To generate a spatial map of the spin-wave amplitude and for dynamic visualization of spin-wave propagation, we capture Faraday rotation images at three different phase settings of the radiofrequency excitation signal (0°, 120°, 240°) and fit a sine function to each location in the image. This measurement procedure is justified by working in the linear regime.

## Online content

Any methods, additional references, Nature Portfolio reporting summaries, source data, extended data, supplementary information, acknowledgements, peer review information; details of author contributions and competing interests; and statements of data and code availability are available at 10.1038/s41563-025-02282-y.

## Supplementary information

Supplementary Information Supplementary Figs. 1–9, Table 1 and Discussion.

## Source data

Source Data Fig. 2 Faraday rotation, transmission and mode-filtered data.

Source Data Fig. 3 Dispersion relation data.

Source Data Fig. 4 Fourier amplitude data plotted in Fig. 4a and Fourier amplitude profiles and transmission data plotted in Fig. 4b.

Source Data Fig. 5 Faraday rotation and transmission data plotted in Fig. 5c,d.

Source Data Fig. 6 Faraday rotation and transmission data.

## Data Availability

The data that support the findings of this study are available from the corresponding author on reasonable request. Source data are provided with this paper.

## Code availability

The code used in this study to generate the simulations is available from the corresponding author on reasonable request.
