# Supplementary material for: Dispersion-tunable low-loss implanted spin-wave waveguides for large magnonic networks
Source: Nat Mater. 2025 Jul 9;24(12):1920–6. doi: 10.1038/s41563-025-02282-y (PMC12657222; doi:10.1038/s41563-025-02282-y)
Supplement: Supplementary file 1 — Supplementary Figs. 1–9, Table 1 and Discussion. [file 41563_2025_2282_MOESM1_ESM.pdf]

nature materials

Supplementary information

https://doi.org/10.1038/s41563-025-02282-y

Dispersion-tunable low-loss implanted spin-wave waveguides for large magnonic networks

In the format provided by the authors and unedited

Table of Contents

1. Stopping range of ions in matter (SRIM) simulations	2
2. Faraday imaging microscope	3
3. Scanning transmission electron microscopy (STEM) measurements	4
4. Simulations of spin-wave propagation in YIG using Mumax^3	4
5. Mechanism of spin-wave confinement in the implanted waveguides	5
6. Frequency-dependent spin-wave decay length in waveguides of different widths	7
7. Transverse spin-wave modes in the waveguides	8
8. Dispersion tuning of waveguides of constant width by implantation	8
9. Peculiarities of the used implantation regime	9
10. Layout of the spin-wave network	10
11. Spin-wave propagation lengths in spin-wave waveguides	10

1. Stopping range of ions in matter (SRIM) simulations

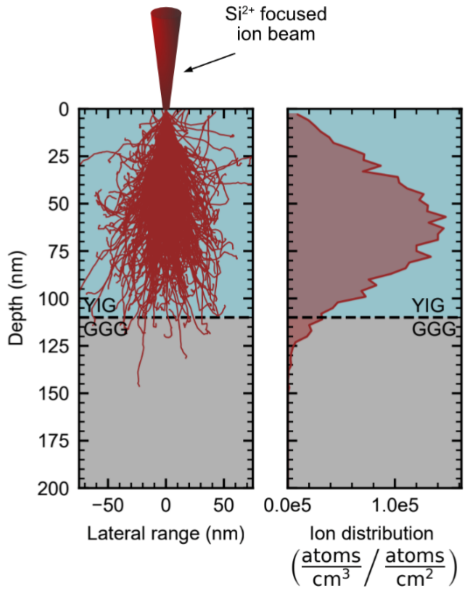

Fig. S1 SRIM simulations for Si^{2+} ions in YIG/GGG.

In order to understand the implantation process of Si^{2+} ions into the YIG film, SRIM simulations are carried out (see Fig. S1).[1] A density of 5.17 g/cm^3 is used for YIG ($Y_3Fe_5O_{12}$) and 7.08 g/cm^3 for GGG ($Gd_3Ga_5O_{12}$). The parameters of the individual elements in the material are specified by the SRIM software and are not changed. An acceleration voltage of 35 kV is used in the focused ion beam machine (Raith Velion) to accelerate doubly charged Si ions, resulting in a kinetic energy of 70 keV. To obtain the depth profile of the implantation, a total number of 5 000 ions is simulated. The trajectories of the ions in the YIG film colliding with the atoms in the material are calculated. 500 of these trajectories are shown as an example in Fig. S1, visualizing the cone-shaped implantation profile. We find that the focused ion beam penetrates the entire 110 nm thick YIG film, with the highest ion implantation density at a depth of 60 nm (Fig. S1).

2. Faraday imaging microscope

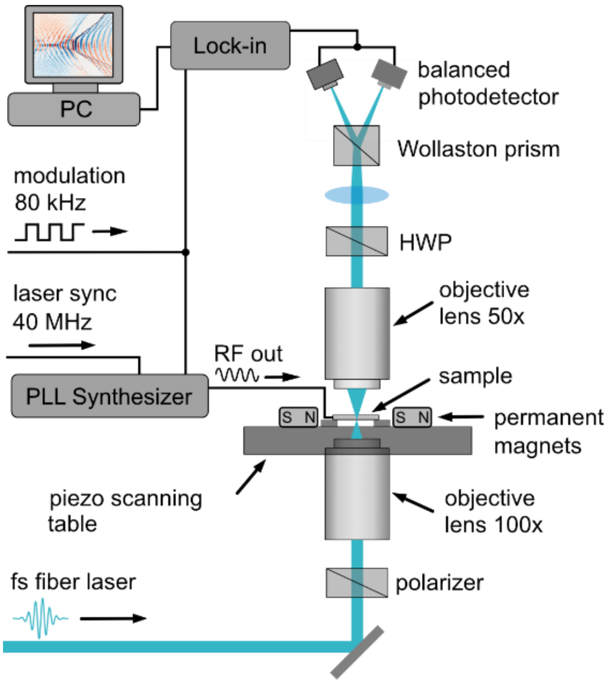

Fig. S2 Schematic drawing of the Faraday imaging setup.

Pulses from a femtosecond fiber laser system at a wavelength of 480 nm are used to image spin waves in YIG. The linear polarization of the laser light transmitted through the sample is rotated due to the out-of-plane magnetization component of the spin waves via the Faraday effect. The rotation is measured with a combination of a half-wave plate, Wollaston prism, and a homebuilt two-channel photodiode detector allowing the measurement of the difference and sum of the intensities in the two channels. Here, the sum signal I_{sum} yields an optical transmission image, while the Faraday rotation angle θ can be calculated from both signals using:

$$2\theta = \arcsin \frac{I_{\mathrm{diff}}}{I_{\mathrm{sum}}}$$

The sample is mounted on an XYZ piezo scanning table (Physik Instrumente PInano XYZ) and placed in an in-plane bias magnetic field of 50 mT, created by a pair of permanent magnets. Microwaves, exciting the spin waves are created by scaling the laser repetition frequency up to the GHz range using a phase-locked loop (PLL) synthesizer. To extract the magnon-induced polarization change, the microwave excitation is modulated with a frequency of 80 kHz, and the received signal is measured at the same frequency using a lock-in amplifier (Stanford Research Systems SR830).

3. Scanning transmission electron microscopy (STEM) measurements

TEM lamellae are prepared from implanted spin-wave waveguides using the focused ion beam (FIB) technique with a ZEISS Crossbeam 540. This system is equipped with a multi-gas injection system and a Ga ion source for milling and thinning the samples to a sub-100 nm thickness, ensuring electron transparency. To enhance stability during the FIB milling process, gold is sputtered on the sample using physical vapor deposition and, to keep the lamella from damage during the thinning process, a protective layer of carbon is deposited atop the lamella. The cross-sectional profile of the exposed regions is imaged using scanning transmission electron microscopy (STEM) with an FEI Titan Themis 60-300 microscope. This microscope features a high-brightness field emission gun (X-FEG) operating at 3.45 kV acceleration voltage and 300 kV extraction voltage, a monochromator, a quadrupole EDS system, an HAADF detector (Fischione Model 3000) and a fast CMOS camera (Ceta, 4kx4k).
The amorphous and crystalline regions, as well as their interfaces, are analyzed using nanobeam diffraction (NBD) mapping, which provides nanometer-scale precision reconstructed structural maps[2,3] using the microprobe STEM mode with a 1 nm probe size and a step size of less than 4 nm in each direction. Data are acquired from each waveguide using drift-corrected spectrum imaging. A custom code written in Digital Micrograph is employed to reconstruct structural maps.

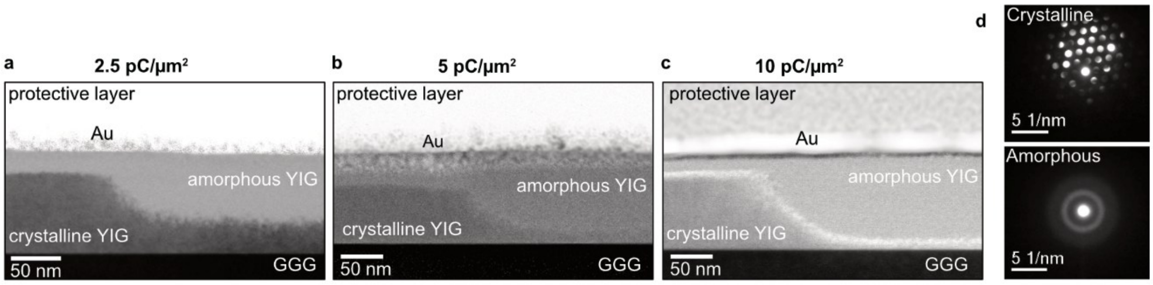

Fig. S3 a-c Measured STEM profiles of cross sections of waveguide edges, implanted with 2.5 pC/µm^2, 5 pC/µm^2 and 10 pC/µm^2, respectively. The thickness of the amorphous YIG increases with increased implantation dose. **d** Nanobeam diffraction patterns of crystalline YIG (top) and amorphous YIG (bottom) regions.

4. Simulations of spin-wave propagation in YIG using Mumax^3

Simulations of waveguide dispersion relations are performed using the mumax^3 package. For the pristine YIG film of 110 nm thickness, we use the following parameters: Saturation magnetization $M_{\text{sat}} = 155\ \text{kA/m}$, exchange constant $A_{\text{exchange}} = 3.75\ \text{pJ/m}$, and damping $\alpha = 7.5 \cdot 10^{-4}\text{m}^{-1}$. For the amorphized YIG, we use $M_{\text{sat}} = 0\ \text{kA/m}$, $A_{\text{exchange}} = 0\ \text{pJ/m}$ and the value for α is unchanged.
To excite spin-waves and extract the dispersion relation, we simulate the antenna by applying an AC magnetic field, which shape corresponds to that of a current-carrying wire placed 50 nm above the waveguide. This excitation field is described by a sinc function with a maximum frequency of $10\ \text{GHz}$ and an amplitude small enough to stay in the linear regime. To create the surface wave configuration, we apply an additional in-plane bias field of $50\ \text{mT}$ along the antenna direction. We run the simulations on a 3D grid with a cell size of $10\ \text{nm}$ x $10\ \text{nm}$ x $11\ \text{nm}$. Using these parameters, we achieve a convincing agreement between experiment and simulation of the dispersion relation of the pristine YIG film (cf. Fig. 3 in the main text).

5. Mechanism of spin-wave confinement in the implanted waveguides

We demonstrate the mechanism of spin-wave confinement using an example of a waveguide with a nominal width of 750 nm (the real width is 650 nm due to the spreading of ions) magnetized by an external magnetic field of 50 mT. The ion implantation amorphizes parts of the crystalline YIG, in which the saturation magnetization M_{sat} is reduced to (almost) zero. The corresponding magnetization drop on both sides of the waveguide causes demagnetizing fields effects.[4] Fig. S4 shows the demagnetizing field (middle column) and effective field (right column) obtained by micromagnetic simulations for different amorphization depths. Even if only the top 20 – 30 % fraction of the total film thickness is amorphized (i.e., a 70 – 80 % pedestal remains), a notable demagnetizing field builds up. The effective magnetic field exhibits a peculiar dependence on the *y* coordinate, perpendicular to the waveguide axis x (Fig. S4b). However, along the *z* direction, the demagnetizing field is almost uniform, even if the amorphization depth is less than 50 % of the total thickness (Fig. S4a, middle column).

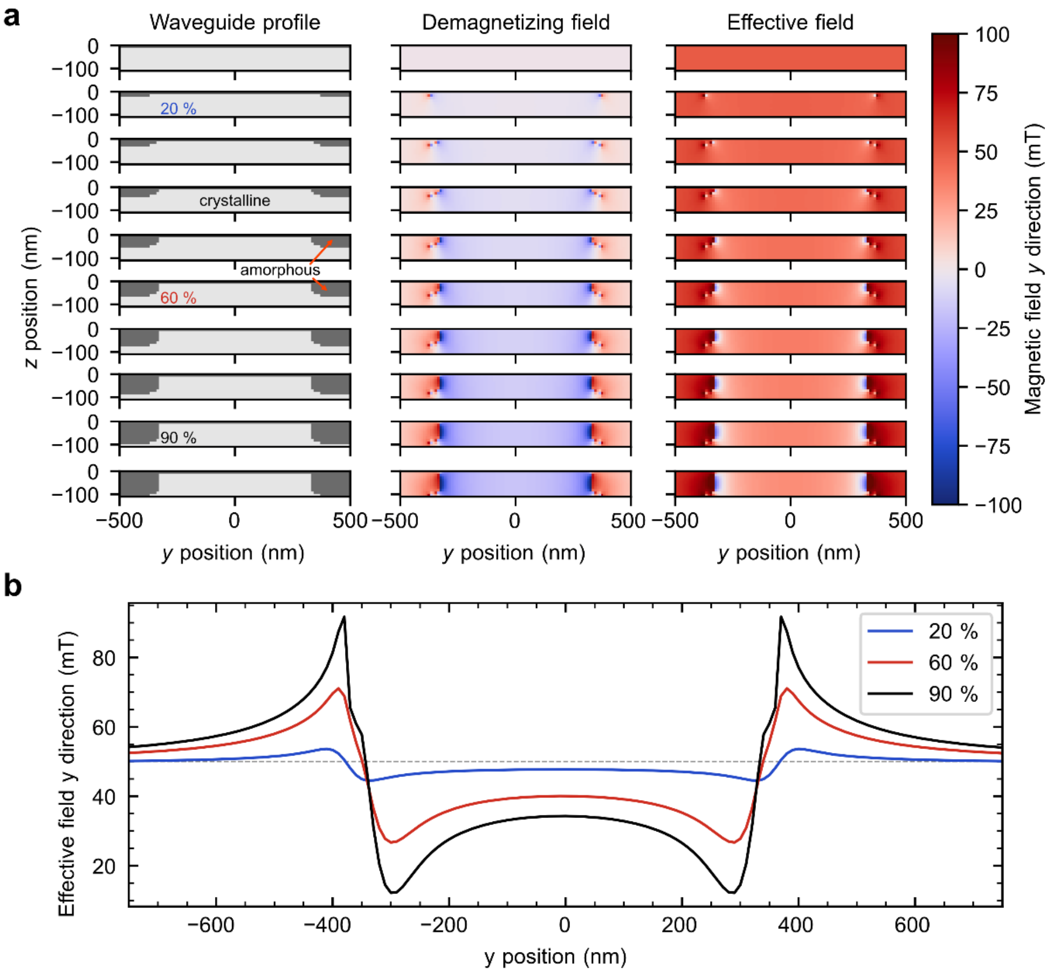

Fig S4 a Calculated demagnetizing field and effective magnetic field in the waveguides for different pedestal heights. The fields are obtained from micromagnetic simulations using the waveguide profiles shown in the left column. The color coding always shows the *y* component of the field, which is parallel to the applied external bias field of 50 mT. **b** Effective magnetic field along a horizontal cut in **a**, at a *z* position centered in the remaining crystalline pedestal.

This demagnetizing field leads to the appearance of confined spin-wave modes in the waveguide region. For an amorphization depth of 90 % (corresponding to our highest implantation dose of 10 pC/µm^2), the effective magnetic field at the center of the waveguide decreases to about 34 mT. As a result of this lower field, the dispersion relation of the spin waves in the waveguide shifts significantly towards smaller frequencies compared to the continuous pedestal film, where the effective field is ≥ 50 mT. Due to this frequency and momentum mismatch, the wave becomes confined in the waveguide: the dynamic susceptibility in the pedestal film at the frequency of the spin wave is small, which results in negligible amplitudes of the dynamic magnetization outside the waveguide (see the corresponding profile of the dynamic magnetization in Fig. S5c).

When the depth of the amorphization decreases to 60 % (corresponding to our lowest implantation dose of 2.5 pC/µm^2), the effective magnetic field at the center of the waveguide increases to about 40 mT. This leads to a decrease in the frequency mismatch and an increase in dynamic susceptibility in the pedestal film at the frequency of the waveguide mode. Accordingly, the profile of the dynamic magnetization exhibits tails evanescently extending into the pedestal (see the profile in Fig. S5b). We emphasize, however, that the dynamic magnetization in the pedestal is still driven in a non-resonant regime. Therefore, the amplitudes of the tails remain small and the profile of the dynamic magnetization inside the waveguide remains almost unchanged.

This trend continues with the decrease of the amorphization depth up to about 20 %. Only at this height does the spatial modulation of the effective field become weak enough (Fig. S4) to ensure matching of the spin wave frequencies inside the waveguide and in the surrounding pedestal film. As a result, the spatial profile of the dynamic magnetization changes dramatically (Fig. S5a): the spin waves become delocalized, and the wave process occupies both the waveguide and the pedestal.

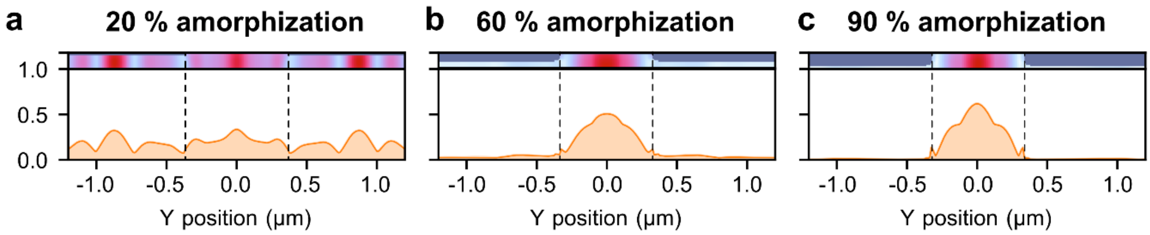

Fig S5 a-c Calculated spin-wave amplitude profiles of the n=1 mode for different amorphization depths of (20 %, 60 % and 90 %). Grey areas denote the amorphized areas in the Y-Z plane. For 20 % amorphization depth, the spin-wave modes are only weakly confined and extend into the pedestal region. For 60 % amorphization depth, spin waves are mostly confined by the implantation, while only a small fraction of the spin waves extends into the pedestal. For 90 % amorphization depth, the spin waves are fully confined in the waveguide defined by ion implantation.

6. Frequency-dependent spin-wave decay length in waveguides of different widths

In general, the spin-wave decay length in the waveguides depends on frequency (Fig. S6).

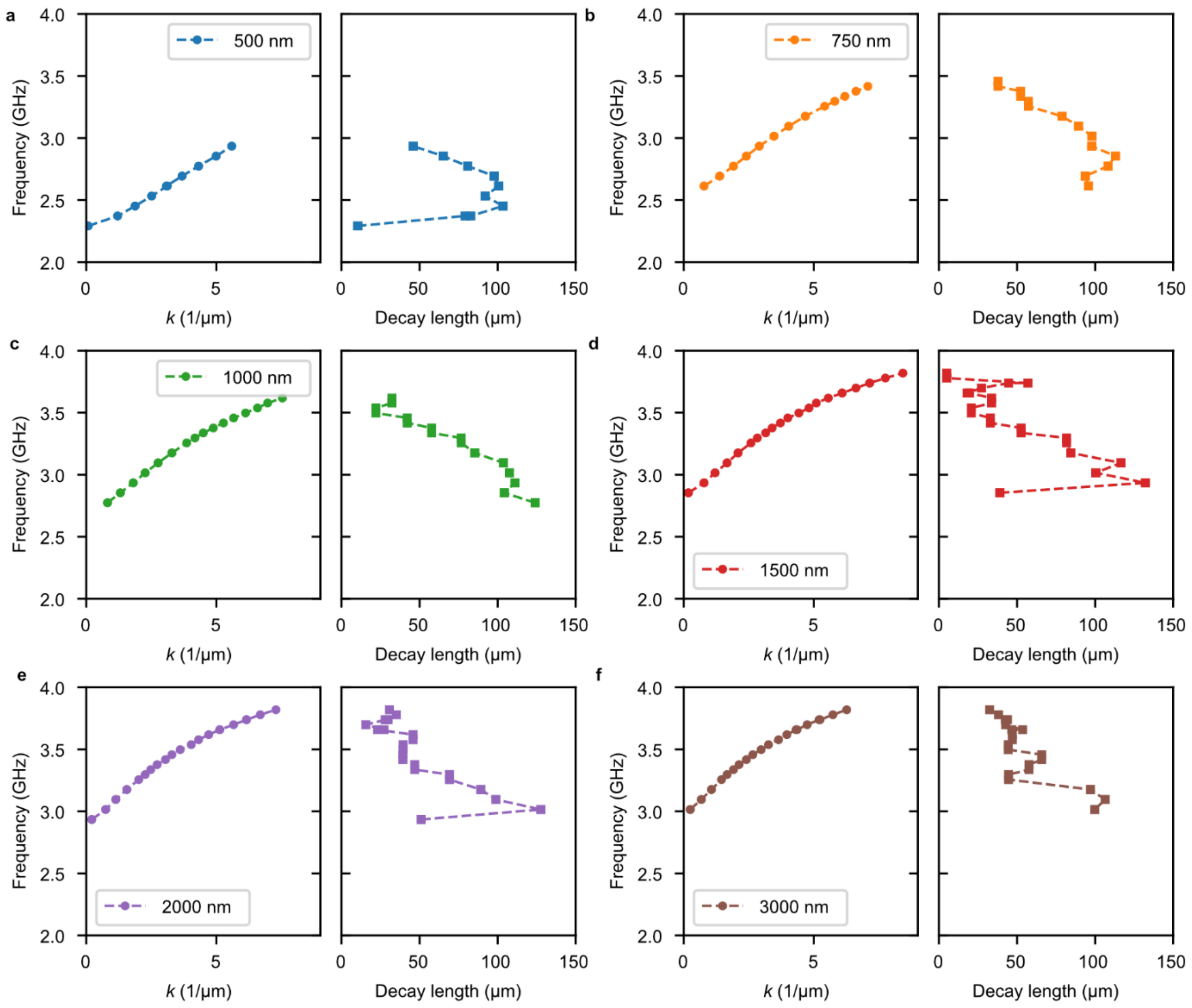

Fig. S6 a-f, Measured dispersion curves (spheres) and frequency-dependent decay lengths (squares) for waveguides of different nominal widths, fabricated with an implantation dose of 2.5 pC/µm². For the larger waveguides (≥ 1 µm), we observe the highest decay lengths for small frequencies and small wave numbers. For higher frequencies, the decay lengths continuously drop. This can be attributed to the diminishing group velocity (slope of the dispersion curve) for higher frequencies. The two sub-micrometer waveguides show a slightly flattened dispersion curve at small frequencies and wave numbers. Consequently, they reach the highest decay length at higher wave numbers.

7. Transverse spin-wave modes in the waveguides

Spatial Fourier transforms of the Faraday rotation for higher implantation doses of 5 pC/µm² and 10 pC/µm² (Fig. S7) show that the spin-wave modes extend less into the implanted area compared to the waveguides written with 2.5 pC/µm² (Fig. 4).

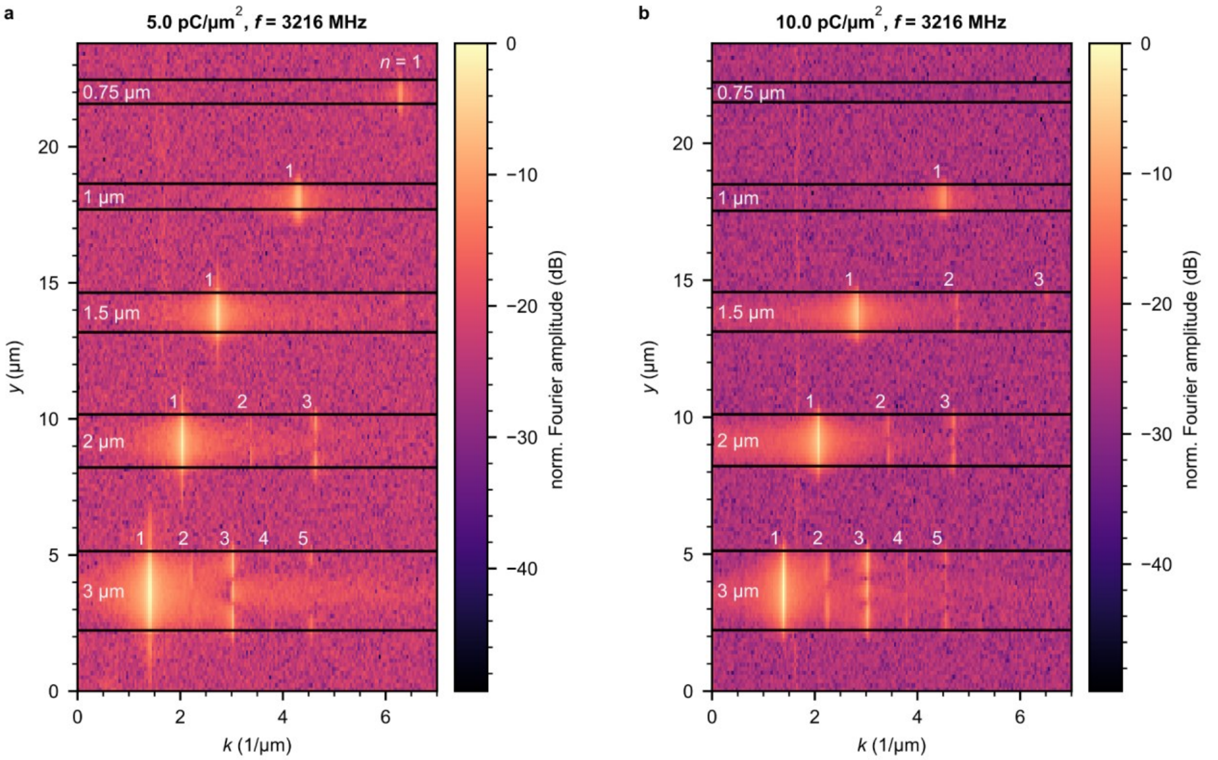

Fig. S7 Line-by-line spatial Fourier transform of the Faraday rotation image using a logarithmic color coding for higher implantation doses of (**a**) 5 pC/µm² and (**b**) 10 pC/µm². Waveguides are marked by black lines and their nominal widths. Higher-order transverse spin-wave modes (n = 2, 3, …) are visible in the wider waveguides.

8. Dispersion tuning of waveguides of constant width by implantation

To further demonstrate the dispersion engineering capabilities offered by ion-implanted spin-wave YIG waveguides, we fabricated a structure shown in Fig. S8, consisting of three linear waveguides with a constant width of 750 nm. A reference waveguide (a) is defined by ion implantation with a dose of 2.5 pC/µm². Waveguide (b) is split up into two parts. While the first part with a length of 50 µm is again implanted with a dose of 2.5 pC/µm², the second is implanted with a dose of 10 pC/µm². Finally, waveguide (c) is created as an alternating dose pattern of 10 µm long areas with doses of 2.5 pC/µm² and 10 pC/µm², respectively. While the spin waves in the homogeneous implanted waveguide regions in a, b and c propagate without change of the wavelength, they exhibit a wavelength shift at each interface between two different implantation doses. Structure c shows that the wavelength can also be switched back by implanting with the dose of the first section again. This type of implanted structure proves the possibility of tuning the wavelength within a linear spin-wave waveguide, which could, for example, be used to generate phase delays between two waveguides without having to geometrically lengthen one of the waveguides.

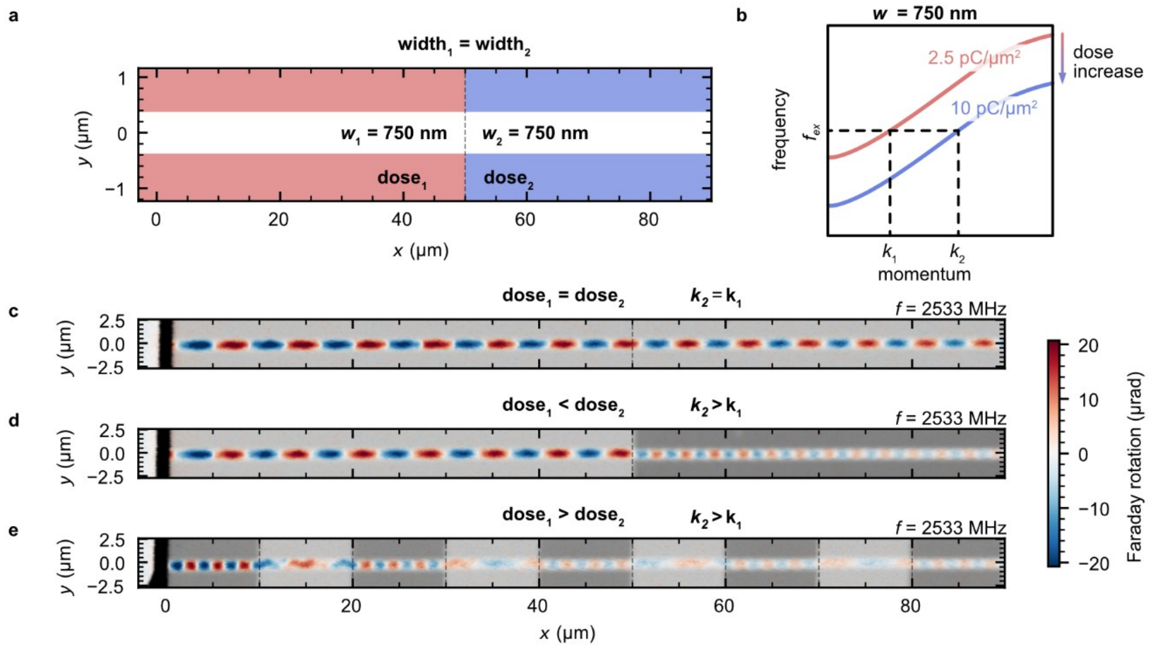

Fig. S8 Dispersion engineering of the spin-wave propagation in linear waveguides with a constant width of 750 nm formed by ion-implanted areas with different doses. **a,** Schematic illustration of the investigated structure. The waveguide is formed by ion-implanted areas with $dose_1$ and $dose_2$, respectively. **b**, Schematic dispersion relations of spin waves propagating in 750 nm wide waveguides with implantation doses of 2.5 pC/µm² and 10 pC/µm². At a certain frequency f_{ex} a spin wave can be excited in the waveguides with both implantation doses but at different wave vectors k. **c-d**, Spin-wave propagation visualized by Faraday rotation. The superimposed optical transmission images indicate the implantation doses (gray scale). **c,** Waveguide with a homogeneous implantation dose of 2.5 pC/µm². **d,** Waveguide with a dose of 2.5 pC/µm² for 0 µm < x < 50 µm and a dose of 10 pC/µm² for x > 50 µm. **e,** Waveguide with an alternating dose profile, switching between 2.5 pC/µm² and 10 pC/µm² every 10 µm. Due to the different doses, the spin-wave wavelength is changed at the interfaces.

9. Peculiarities of the used implantation regime

The manipulation of spin waves in YIG by ion implantation depends strongly on the dose, energy and mass of the used ions. Here, one needs to consider three different implantation regimes: (i) Very weak implantation, as used by Kiechle et al.[5] (for example 10^{12} – 10^{13} Ga^+ ions cm^{-2} at 50 keV), slightly alters the saturation magnetization of YIG and enables diffractive spin-wave optics. These implantation doses do not amorphize the crystalline YIG, but lead to an implantation of dopants into the crystalline YIG structure. (ii) Stronger implantation with Ga^+ ions at 50 keV (dose 10^{13} - 10^{14} ions cm^{-2}) amorphize only the top layer (~ 25 nm) of the YIG film due to the limited penetration depth of the Ga^+ ions at this kinetic energy. This regime effectively decreases the thickness of the YIG film, thereby changing its dispersion relation.[6] However, this amorphization fraction is not effective for the creation of spin-wave waveguides, due to insufficient lateral confinement. (iii) High implantation doses as used in this work (> 10^{15} Si^{2+} ions cm^{-2} at 70 keV), amorphize a larger fraction of the YIG film. Here, neither the ion species nor the density of the implanted ions plays an important role, except that the ion mass and kinetic energy determine the amorphization depth and profile. The fraction of the amorphization next to the waveguide determines primarily the demagnetizing field inside the waveguide, effectively leading to the confinement of spin waves in the

waveguides, while suppressing extended film modes. In this regime, no spin waves propagate in the implanted areas.

10. Layout of the spin-wave network

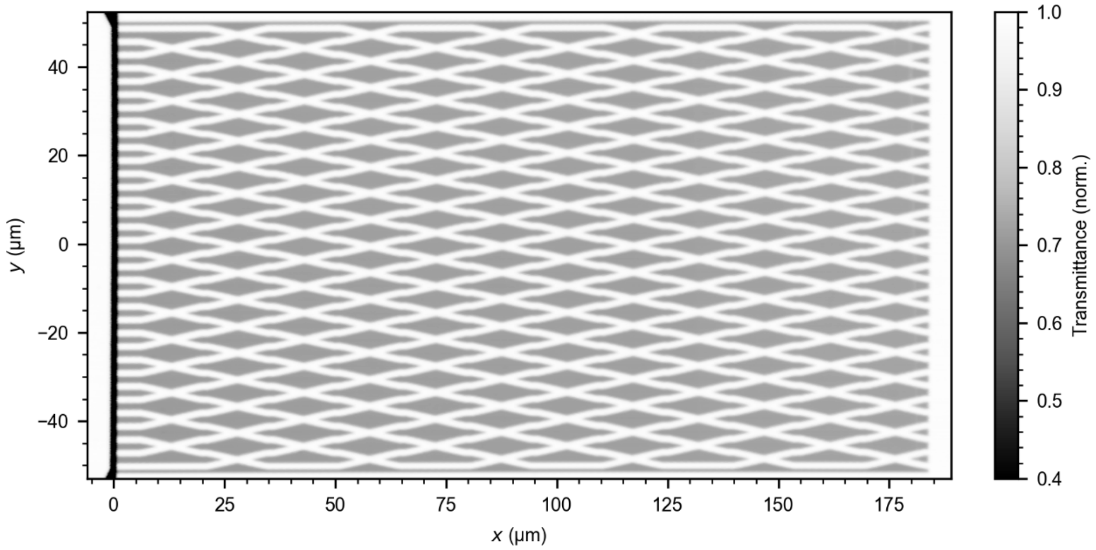

Fig. S9 Optical transmission image of the fabricated spin-wave network.

11. Spin-wave propagation lengths in spin-wave waveguides

Table S1 Comparison of spin-wave propagation lengths in spin-wave waveguide (WG) systems based on pristine or hybrid YIG waveguides.

Type of waveguides	Growth and processing techniques	YIG thickness (nm)	WG width (µm)	Film parameters	Decay length (µm)
**YIG/GGG [this work]**	LPE, FIB	110	0.5 to 3	FMR linewidth < 2 Oe	**> 100**
**YIG/GGG [7]**	PLD, lithography	80	0.5	N/A	54
**YIG/GGG [8]**	PLD, etching	20	2.5	FMR linewidth : 1.9 Oe at 6GHz, 1.4 Oe at zero frequency<br>α =4 x 10^{-4}<br>M_{sat} = 169.5 kA/m	25
**YIG/GGG [9]**	Sputtering, lithography	40	4	FMR linewidth : 1.03 mT at 15 GHz<br>α =2.77±0.49 x 10^{-4} M_{sat} = 1122±0.3 kA/m	10
**La:YIG/GGG [10]**	LPE, masking	44	1<br>0.3<br>0.05	FMR linewidth : 0.18± 0.01 mT<br>α =1.75±0.08 x 10^{-4}<br>M_{sat} = 140.7±2.8 kA/m	12<br>4.6<br>1.8

**Au/Pt/YIG/GGG** [11]	Sputtering, etching	49	14	FMR linewidth : 0.3 ± 0.01 mT at zero frequency<br>α =(2.4 ± 0.3) x 10^{-4}<br>M_{sat} = 127± 7 kA/m	3.6
**Pt/YIG/GGG** [12]	LPE, sputtering, etching	100	5	FMR linewidth : 2 Oe at 3.5 GHz, 1.6 Oe at zero frequency<br>α =2.8 x 10^{-4}<br>M_{sat} = 144±2 kA/m	31
**CoFeB/TaO/YIG/GGG** [13]	PLD, sputtering, lithography	66	0.16	α =5.2±1.1 x 10^{-4}<br>M_{sat} = 181 ± 5 kA/m	~20

References:

[1] J.F. Ziegler, M.D. Ziegler, and J.P. Biersack, "SRIM – The stopping and range of ions in matter (2010)," Nuclear Instruments and Methods in Physics Research Section B: Beam Interactions with Materials and Atoms **268**(11), 1818–1823 (2010).

[2] C. Gammer, V. Burak Ozdol, C.H. Liebscher, and A.M. Minor, "Diffraction contrast imaging using virtual apertures," Ultramicroscopy **155**, 1–10 (2015).

[3] V.B. Ozdol, C. Gammer, X.G. Jin, P. Ercius, C. Ophus, J. Ciston, and A.M. Minor, "Strain mapping at nanometer resolution using advanced nano-beam electron diffraction," Applied Physics Letters **106**(25), 253107 (2015).

[4] V.E. Demidov, and S.O. Demokritov, "Magnonic Waveguides Studied by Microfocus Brillouin Light Scattering," IEEE Transactions on Magnetics **51**(4), 1–15 (2015).

[5] M. Kiechle, A. Papp, S. Mendisch, V. Ahrens, M. Golibrzuch, G.H. Bernstein, W. Porod, G. Csaba, and M. Becherer, "Spin-Wave Optics in YIG Realized by Ion-Beam Irradiation," Small **19**(21), 2207293 (2023).

[6] J. Greil, M. Kiechle, A. Papp, P. Neumann, Z. Kovács, J. Volk, F. Schulz, S. Wintz, M. Weigand, G. Csaba, and M. Becherer, "The effect of Ga-ion irradiation on sub-micron-wavelength spin waves in yttrium-iron-garnet films," Nanotechnology **36**(13), 135301 (2025).

[7] K.O. Nikolaev, S.R. Lake, G. Schmidt, S.O. Demokritov, and V.E. Demidov, "Resonant generation of propagating second-harmonic spin waves in nano-waveguides," Nat Commun **15**(1), 1827 (2024).

[8] M. Collet, O. Gladii, M. Evelt, V. Bessonov, L. Soumah, P. Bortolotti, S.O. Demokritov, Y. Henry, V. Cros, M. Bailleul, V.E. Demidov, and A. Anane, "Spin-wave propagation in ultra-thin YIG based waveguides," Applied Physics Letters **110**(9), 092408 (2017).

[9] M.B. Jungfleisch, W. Zhang, W. Jiang, H. Chang, J. Sklenar, S.M. Wu, J.E. Pearson, A. Bhattacharya, J.B. Ketterson, M. Wu, and A. Hoffmann, "Spin waves in micro-structured yttrium iron garnet nanometer-thick films," Journal of Applied Physics **117**(17), 17D128 (2015).

[10] B. Heinz, T. Brächer, M. Schneider, Q. Wang, B. Lägel, A.M. Friedel, D. Breitbach, S. Steinert, T. Meyer, M. Kewenig, C. Dubs, P. Pirro, and A.V. Chumak, "Propagation of Spin-Wave Packets in Individual Nanosized Yttrium Iron Garnet Magnonic Conduits," Nano Lett. **20**(6), 4220–4227 (2020).

[11] A. Talalaevskij, M. Decker, J. Stigloher, A. Mitra, H.S. Körner, O. Cespedes, C.H. Back, and B.J. Hickey, "Magnetic properties of spin waves in thin yttrium iron garnet films," Phys. Rev. B **95**(6), 064409 (2017).

[12] P. Pirro, T. Brächer, A.V. Chumak, B. Lägel, C. Dubs, O. Surzhenko, P. Görnert, B. Leven, and B. Hillebrands, "Spin-wave excitation and propagation in microstructured waveguides of yttrium iron garnet/Pt bilayers," Applied Physics Letters **104**(1), 012402 (2014).

[13] H. Qin, R.B. Holländer, L. Flajšman, and S. van Dijken, "Low-Loss Nanoscopic Spin-Wave Guiding in Continuous Yttrium Iron Garnet Films," Nano Lett. **22**(13), 5294–5300 (2022).
